# Usefulness of Amino Acid Profiling in Ovarian Cancer Screening with Special Emphasis on Their Role in Cancerogenesis

**DOI:** 10.3390/ijms18122727

**Published:** 2017-12-16

**Authors:** Szymon Plewa, Agnieszka Horała, Paweł Dereziński, Agnieszka Klupczynska, Ewa Nowak-Markwitz, Jan Matysiak, Zenon J. Kokot

**Affiliations:** 1 Department of Inorganic and Analytical Chemistry, Poznan University of Medical Sciences, 6 Grunwaldzka Street, 60-780 Poznan, Poland; szymonplewa1@gmail.com (S.P.); p.derezinski@gmail.com (P.D.); aklupczynska@ump.edu.pl (A.K.); jmatysiak@ump.edu.pl (J.M.); 2 Gynecologic Oncology Department, Poznan University of Medical Sciences, 33 Polna Street, 60-535 Poznan, Poland; agnieszka0lemanska@gmail.com (A.H.); ewamarkwitz@ump.edu.pl (E.N.-M.)

**Keywords:** amino acids, targeted metabolomics, ovarian cancer, biomarkers, screening, metabolic pathways analysis

## Abstract

The aim of this study was to quantitate 42 serum-free amino acids, propose the biochemical explanation of their role in tumor development, and identify new ovarian cancer (OC) biomarkers for potential use in OC screening. The additional value of this work is the schematic presentation of the interrelationship between metabolites which were identified as significant for OC development and progression. The liquid chromatography-tandem mass spectrometry technique using highly-selective multiple reaction monitoring mode and labeled internal standards for each analyzed compound was applied. Performed statistical analyses showed that amino acids are potentially useful as OC biomarkers, especially as variables in multi-marker models. For the distinguishing metabolites the following metabolic pathways involved in cancer growth and development were proposed: histidine metabolism; tryptophan metabolism; arginine biosynthesis; arginine and proline metabolism; and alanine, aspartate and glutamine metabolism. The presented research identifies histidine and citrulline as potential new OC biomarkers. Furthermore, it provides evidence that amino acids are involved in metabolic pathways related to tumor growth and play an important role in cancerogenesis.

## 1. Introduction

Ovarian cancer (OC) is one of the most lethal cancers among women in Europe, with the incidence of 13.1 per 100,000 women in 2012 [1,2]. It is related with the death of 42,700 women in Europe yearly [3]. The main reason of high mortality is lack of proper diagnostic tools to detect the disease in early stages. As OC usually develops without well-defined clinical symptoms, it is diagnosed mostly in advanced stages (i.e., stage III-IV according to International Federation of Gynecology and Obstetrics—FIGO), with poor five-year survival rates of 15–45%. [4]. On the other hand, FIGO I stage OC is related with 90% or higher survival rate. In order to improve the diagnostic procedures and identify OC patients in early stages of the disease, the search for its novel specific biomarkers has been ongoing for decades. Until now, several models were proposed for clinical use, e.g., cancer antigen 125 (CA 125), human epididymis protein 4 (HE 4), a combination of those with clinical features or ultrasound imaging, but their sensitivity and/or specificity are still unsatisfactory, especially for early stages of OC [4,5] and no effective screening method has so far been found.

Nowadays “omics” technologies, such as proteomics, metabolomics, and genomics, have emerged as promising tools for biomarkers research. Analyzing the genome, transcriptome, proteome, and metabolome in various biological fluids can reveal changes at different functional levels, associated with the ongoing disease. Among these modern techniques metabolomics seems to be very promising for biomarker research due to dynamic character of metabolome that reflects current processes in the human body and can be used to distinguish between physiological and pathological states.

The identified metabolic disturbances could potentially serve as diagnostic, prognostic, predictive, or even efficacy markers. Recent studies have shown altered concentrations of various metabolites from many different classes in OC patients, such as lysophospholipids [5,6,7], acylcarnitines, and amino acids [8]. Amino acid profile has also been identified as an effective diagnostic tool in different cancers before [9,10,11,12]. Moreover, a systematic review of the results of untargeted metabolomics studies reveals that some amino acids can be associated with OC [13,14,15,16]. However, the untargeted metabolomic experiments fail to provide information about the absolute concentration of analyzed compounds, reporting only the signal intensities, which are difficult to translate into clinical practice.

The aim of this study was to quantitate free amino acid and biogenic amine profile in human serum and identify potential OC biomarkers with a special emphasis on OC screening. Three groups of patients were analyzed: OC, benign ovarian tumor (BOT) and a matched healthy control group (HC), used as a reference group. This is the first study based on targeted metabolomics with such a broad coverage of quantified proteinogenic and non-proteinogenic amino acids in OC, BOT, and HC patients. The presented study was performed based on the up-to-date liquid chromatography/tandem mass spectrometry (LC-MS/MS) technique using highly-selective multiple reaction monitoring (MRM) mode and labeled internal standards for each analyzed compound. In the available literature there is a limited number of studies utilizing both MRM mode and labeled internal standards for metabolomics studies on OC patients’ serum.

Moreover, presented research proposes a possible interrelationship between compounds which were previously found to have altered concentrations in OC patients’ serum.

## 2. Results

### 2.1. Serum-Free Amino Acid Profiles

Amino acid profiles were obtained using validated and robust methodology, enabling quantitating 42 analytes from three groups: proteinogenic amino acids, non-proteinogenic amino acids, and biogenic amines. In the analyzed samples 33 amino acids were determined. For the remaining nine analytes concentration level was lower than the limit of quantitation (LOQ) or concentration exceeded LOQ only for part of the samples—these compounds were excluded from statistical analyses (phosphoserine, homocitrulline, argininosuccinic acid, γ-amino-*n*-butyric acid, anserine, carnosine, δ-hydroxylysine, cystathionine, and homocystine).

### 2.2. Ovarian Cancer versus the Healthy Control Group

**Figure 1:**
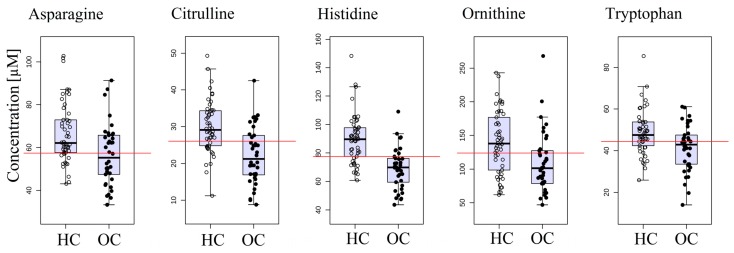
Serum concentrations of 5 amino acids that obtained the lowest *p*-value in the comparison between ovarian cancer (OC) patients and healthy control (HC) group. The optimal cutoff values based on univariate ROC curve analysis are overlaid on the box-plots.

Twelve out of 33 compounds had significantly altered concentrations between OC patients and HC: 1-methyl-l-histidine, alanine, asparagine, citrulline, cystine, ethanolamine, histidine, lysine, methionine, ornithine, threonine, and tryptophan (Table S1). Almost all of the amino acids found as significantly altered were decreased in OC group, with exception of cysteine which level was increased in the OC group. Figure 1 presents box-plots for five amino acids with the lowest *p*-value (*p* < 0.005).

In the next step the univariate receiver operating characteristic (ROC) curve analyses were performed to compare the discriminating capabilities of the 12 selected amino acids. For two of them the area under the curve (AUC) was higher than 0.75: histidine (AUC = 0.836, 95% confidence interval (CI): 0.738–0.909, sensitivity 0.789, specificity 0.760), and citrulline (AUC = 0.768, 95% CI: 0.663–0.873, sensitivity 0.711, specificity 0.720).

Application of partial least squares-discriminant analysis (PLS-DA) resulted in a satisfactory distinction of both groups. This indicated that OC patients had different amino acid profiles compared to healthy women. It is worth emphasizing that the highest values of variable importance in projection (VIP) scores were obtained for histidine (VIP = 2.469) and citrulline (VIP = 1.830) (Figure S1). This is consistent with the results of the univariate ROC curve analyses.

We have decided to evaluate the panel of the selected compounds using stepwise forward discriminant function analysis (DFA). Based on the conducted statistical analyses histidine and ornithine were subjected to DFA. The total group membership classification was 78.41% (model 1) (Table 1).

### 2.3. Ovarian Cancer versus Combined Benign Ovarian Tumor and Healthy Control Groups

**Figure 2:**
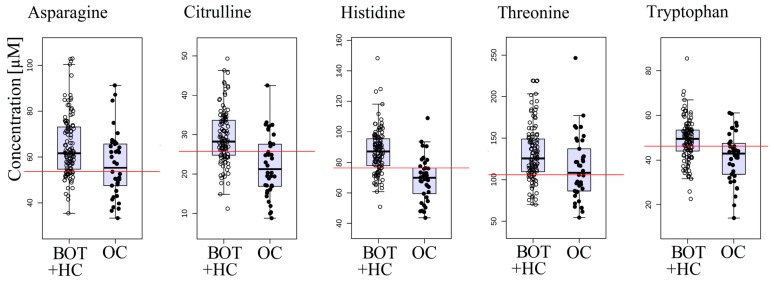
Serum concentrations of 5 amino acids that obtained the lowest *p*-value in the comparison between ovarian cancer (OC) patients and combined benign ovarian tumor (BOT) and healthy control (HC) group. The optimal cutoff values based on univariate ROC curve analysis are overlaid on the box-plots.

The OC group was compared with a combined group consisting of women with BOT and HC in order to verify whether the changes in serum free amino acid profile are caused only by the presence of ovarian malignancy and not by any other pathology and thus better reflected the clinical diagnostic reality of a screening test. Twelve amino acids were found to be significantly altered between the groups: α-aminoadipic acid, asparagine, citrulline, cystine, glutamine, histidine, isoleucine, lysine, methionine, ornithine, threonine, and tryptophan (Table S1). Figure 2 presents the box-plots for five amino acids characterized by the lowest *p*-value. Nine amino acids were the same as for OC versus HC comparison, suggesting that the majority of amino acid profile alterations are specific to malignant (but not benign) ovarian tumors.

This leads to a conclusion that the changes in the amino acid profile are strongly correlated with the development of a malignant tumor. The one-way ANOVA test, used to compare the amino acid profile between the three groups, confirmed our previous observations: significant alterations in the concentrations of histidine and citrulline were found in both comparisons: OC patient versus BOT and OC patient versus HC. Partial least squares-discriminant analysis (PLS-DA) analysis applied for OC versus the combined BOT and HC groups provided sample distinction comparable to OC versus HC patients (Figures S1 and S2). The highest VIP score was obtained for the same amino acids: histidine and citrulline, 2.692 and 2.057, respectively (Figure S2).

For OC versus combined BOT and HC groups stepwise forward DFA was also performed. The following five variables were subjected to the model: histidine, citrulline, alanine, asparagine, and ornithine. The total group membership classification of 86.00% was obtained (model 2) (Table 1). It confirms that the model proposed by us based on selected amino acids provides a satisfactory distinction between the OC patients and the remaining women.

## 3. Discussion

To the best of our knowledge, this is the first study that demonstrates the alterations in concentrations of a wide spectrum of free amino acids in serum of patients with OC, BOT, and HC using a fully-validated, robust quantitative method based on labeled internal standards for each metabolite and MRM mode. The presented study proved that the HPLC-MS/MS-based metabolomic platform is a promising methodology to search for OC biomarkers. Targeted metabolomics is a powerful tool in modern biomarker research due to the possibility of simultaneous analysis of multiple compounds using very small volumes of biological fluids (microliters per analysis). Moreover, defining the metabolomic pattern may provide important information on the mechanism of biological changes in OC, especially in a combination with complementary “omics” sciences. 

Recent evidence indicates that cancer development entails metabolism dysregulation and as a result, leads to changes in the concentrations of various metabolites in biological fluids of cancer patients. Some studies demonstrated that serum or plasma amino acid profiling may be useful in early detection of several cancers [9,11,17,18] and to determine the crucial compounds for OC pathogenesis [5,6,13,19,20] (Table 2).

The aim of the presented study was to identify new potential OC biomarkers and investigate the usefulness of amino acid profiling in OC screening. The novelty of the study involves a wide panel of analyzed metabolites, statistical analyses performed on absolute concentration values, and a combined control group comprising BOT and HC. It should be noted that some studies did not involve the HC group [7,8]. Involvement of the HC group enables comprehensive interpretation of metabolomic alterations as it takes into account the levels of metabolites observed in the general population and is essential to assess the usefulness of a biomarker in screening.

**Figure 3:**
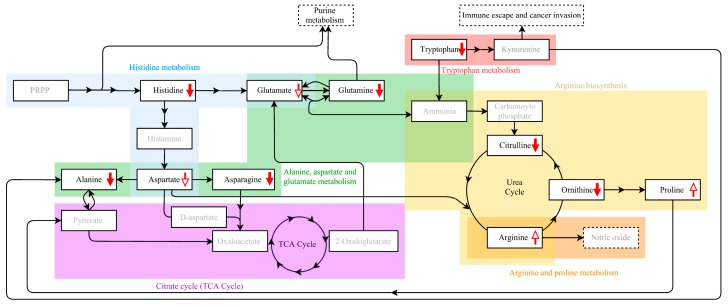
Metabolic pathways proposed to be associated with ovarian cancer (OC) and amino acids altered in OC patients. Red filled arrows represent the concentration of a particular metabolite significantly altered in OC patients (*p*-value < 0.05), red empty arrows indicate the change in concentration of a particular metabolite in OC patients that was not statistically significant (*p*-value > 0.05). The crucial intermediates connecting the proposed amino acids and different metabolic pathways are presented in grey. Dotted rectangles represent the endpoint products, pathways or processes. Abbreviations: PRPP—phosphoribosyl pyrophosphate; TCA—tricarboxylic acid.

Bachmayr-Heyda et al. [13] provided evidence that there is a relationship between amino acids levels and OC metabolism. Our findings also showed significant differences in concentrations of amino acids between OC patients and BOT or HC women. In their research 179 metabolites, including 42 amino acids and biogenic amines, were identified in a semi-quantitative manner. They observed a positive correlation between decreased histidine, lysine, threonine, tryptophan, and citrulline levels and unfavorable prognosis. Although the cited study was limited to high-grade serous OC, it remains in line with our findings, where the levels of these five amino acids were significantly decreased in OC patients compared to HC, as well as compared to combined BOT and HC. Our results provide strong evidence that amino acids can be useful in OC screening, especially as compounds of multi-marker models. Six compounds were identified by Zhang et al. as significantly altered between OC patients and BOT. Among them, a decreased plasma level of the amino acid tryptophan was observed in OC patients. A correlation between tryptophan degradation by malignant tumor cells and indoleamine-(2,3)-dioxygenase (IDO) enzyme was found. IDO is potentially related to “omitting” the human immune system by the tumor cells [7]. Tryptophan degradations by IDO leads to increased kynurenine formation (Figure 3), which causes suppression of T lymphocytes and natural killer cell (NK cell) proliferation [21,22]. It is potentially consistent with our findings where tryptophan was found to be significantly decreased in OC patients’ serum compared to HC, as well as compared to combined HC and BOT. However, the performed univariate ROC curve analyses failed to provide satisfactory AUC values for that amino acid (AUC values ≤ 0.75). The ROC curves are one of the crucial tests used by clinicians for evaluation of biomarker performance. In medical diagnostics, very high AUCs are strongly eligible. In our analysis we recognized the AUC ≥ 0.75 as important, based on widely acceptable rules, that 0.7 ≤ AUC < 0.8 denotes an acceptable discrimination or fair test. Each higher value means improved discriminatory ability of the proposed model. As concluded by Carter et al.: “a model would be considered reasonable with a [AUC] value of >0.7, and strong when ≥0.8” [23], but it should be noted that in clinical practice AUC ≥ 0.9 is extremely unusual [24]. Thus, based on our results, it can be concluded that tryptophan is not sufficiently sensitive to be considered as a potential OC biomarker, in contrast to histidine and citrulline.

We observed decreased levels of serum histidine in OC patients compared to both BOT and HC group. Decreased histidine level have also been previously reported, e.g., for cervical cancer [11] and for lung cancer [9,17]. It was suggested that this resulted from an increased histidine turnover associated with active nucleic acid metabolism in the tumor cells. Zhang et al. [19] have also observed down-regulated urinary histidine in epithelial ovarian cancer patients. Amino acid histidine is postulated to be related with the pathways of nucleotide biosynthesis through 5-phosphoribosyl-1-pyrophosphate (PRPP), which is the crucial substrate of histidine, tryptophan, purine and pyrimidine nucleotides biosynthesis. The de novo synthesized nucleotides are necessary to ensure efficient DNA replication and increased RNA synthesis in fast-growing tumor cells [25]. These findings strongly suggest a potential role of histidine in cancer growth and development. Amino acid glutamine alongside PRPP is also engaged in nucleotide metabolism, which is upregulated in tumor cells. It might serve as a nitrogen donor for purine and pyrimidine biosynthesis, as well as to provide Krebs cycle (TCA Cycle) intermediates during nucleotide biosynthesis, e.g., by glutaminolysis process [26]. Previous research has also demonstrated a correlation between glutamine metabolism and tumor suppressors (p53) and oncogenes (c-myc) proving cancer-glutamine dependency [27]. In our study glutamine level was decreased in OC patients’ serum compared to HC (Table S1). This observation is consistent with the aforementioned studies and might prove increased glutamine turnover in cancerogenesis. Other potential mechanism which connects glutamine levels and cancerogenesis is the link between glutamine and citrulline (Figure 3). Conversion of glutamine in enterocytes is one of the most important sources of circulating citrulline [28]. The circulating citrulline is involved in three metabolic pathways: arginine biosynthesis, the arginine-citrulline-nitric oxide cycle, and the urea cycle (Figure 3). It is worth emphasizing that citrulline is a substrate of arginine biosynthesis and a product of its metabolism [28]. In turn, arginine and nitric oxide have been proved to have a well-established role in tumor development by the impact on tumor angiogenesis, proliferation, and metastasis [29]. Our findings imply that amino acids play a crucial role in the human body not only as building blocks or nutrients, but also as important intermediates in metabolic pathways involved in cancerogenesis. Hence, amino acids should be intensively examined as potential clinical diagnostic biomarkers or efficacy biomarkers for monitoring cancer treatment targeted on specific metabolic pathway.

An additional advantage of the presented study is the evaluation of utility of various amino acids in prediction models using stepwise forward discriminant function analysis (DFA) for OC versus HC, as well as OC versus combined BOT and HC. For the former comparison the highest sensitivity (76.32%, Table 1) was obtained for a model consisted of two predictors: histidine and ornithine. Total group membership classification reached 78.41%. For the latter comparison, OC vs. combined BOT and HC, the highest sensitivity (60.53%) was obtained by the model with five variables (histidine, citrulline, alanine, asparagine, ornithine) with the total group membership classification of 86.00%. Results of the performed DFA for both types of comparisons depict that the sensitivity, which shows the percentage of positives that are properly identified as such, is significantly lower in the case of OC vs. combined BOT and HC compared with OC vs. HC in spite of the higher number amino acids used in the model. This clearly shows that including BOT in the analysis makes the selection of OC more difficult and could lead to a conclusion that the presence of BOT also affects the amino acid profile. Nevertheless, amino acids could potentially be good clinical biomarkers of OC, especially as components of multi-marker models with other substances.

There are limitations in the presented study. First of all, we have only focused on OC and did not examine if other types of cancer may induce similar metabolic alterations. Due to a limited number of samples in each group, the study should be regarded as a pilot study. In further studies special attention should be paid to increase the number of patients, possibly to perform a multicenter study to ensure independent patient cohorts with different types of cancer. Metabolic alterations might be caused, e.g., by ovarian malignancy only, or malignant transformation processes in general. In order to verify the genesis of these alterations studies involving different types of cancer should be performed.

## 4. Materials and Methods

### 4.1. Patients and Sample Collection

Serum samples were collected in the Department of Gynecologic Oncology, Poznan University of Medical Sciences, in accordance with the Declaration of Helsinki, after approval of the study protocol and of the written information for patients by the Local Bioethical Committee of Poznan University of Medical Sciences, Poland (decision no. 165/16 of 4 February 2016 and 80/17 of 5 January 2017). All participants were familiar with the goals of the study and signed the informed consent prior the enrollment. After analyzing the exclusion criteria which were: presence of OC other than epithelial, presence of other cancers, or cancer treatment (radiotherapy, chemotherapy, hormonal therapy) at the moment of sample collection or before, presence of chronic metabolic diseases (diabetes, dyslipidemia, metabolic syndrome), relevant concomitant medication (anti-diabetic agents, statins, hormonal replacement therapy, oral contraception), a total of 150 samples qualified for the study were divided into three groups: 38 patients with epithelial OC, 62 patients with BOT and 50 HC patients. The HC group involved women who underwent surgical intervention for reasons other than ovarian pathology. All three groups were matched in terms of age, BMI and ethnicity (Caucasian). All serum samples were collected by the same physician on the day of the operation after overnight fasting and in the same manner (incubated for 30 min in room temperature for clotting, centrifuged for 15 min at 4000 rpm at 4 °C, aliquoted, and stored at −80 °C in identical vials). Detailed characteristics of all subjects are summarized in Table S2.

### 4.2. Determination of Free Amino Acid Profiles

For quantitation of analytes the aTRAQ kit for amino acid analysis (SCIEX, Framingham, MA, USA) was used. In addition to amino acids the applied methodology enabled the quantitation of several biogenic amines. Prior to the LC-MS/MS assays, sample preparation was performed according to the protocol provided by the manufacturer. Firstly, 40 μL of serum was mixed with sulfosalicylic acid to precipitate proteins. Next the supernatant was diluted with labeling buffer (pH = 8.5). The sulfosalicylic acid and labeling buffer were enriched with non-physiological amino acids norleucine and norvaline, respectively. These amino acids were added for the purpose of quality control, in particular for labeling efficiency evaluation. Then, the amino acid labeling with aTRAQ Reagent Δ8 was performed. Labeling was stopped by addition of hydroxylamine. Next, the internal standard solution was added to the reaction mixture. In the next step the samples were concentrated in a vacuum concentrator (miVac Duo, Genevac, Stone Ridge, NY, USA). Concentrated samples were diluted with 20 μL of water and transferred to autosampler vials with low-volume inserts.

LC-MS/MS assays were performed using a 1260 Infinity HPLC instrument (Agilent Technologies, Santa Clara, CA, USA) coupled with a 4000 QTRAP mass spectrometer (SCIEX, Framingham, MA, USA). For amino acids separation Sciex C18 column (4.6 mm × 150 mm, 5 μm) maintained at 50 °C, and binary gradient at the flow rate 800 μL/min were used. The mobile phase was 0.1% formic acid (FA) and 0.01% heptafluorobutyric acid (HFBA) in water—phase A, and 0.1% FA and 0.01% HFBA in methanol—phase B.

The 4000 QTRAP mass spectrometer was operating in MRM mode using the scheduled MRM algorithm to improve quality and reproducibility of the assays. Data acquisition and management were performed under control of Analyst 1.5.2 software (Sciex, Framingham, MA, USA). Method validation, detailed sample preparation process and LC-MS/MS parameters were described by us before [30]. All samples were prepared and analyzed in a random order.

### 4.3. Statistical Analysis

The statistical analysis and data visualization were performed using STATISTICA 12.5 software (StatSoft, Kraków, Poland) and the MetaboAnalyst web server [31] (www.metaboanalyst.ca). The Shapiro-Wilk's test for the assessment of normality of variables was applied. For normally-distributed free amino acid concentrations the Levene’s test was performed to verify the equality of variances. If variances were equal, the *t*-test was used for statistical significance assessment. Otherwise, unequal variances *t*-test (Welch's *t*-test) was applied. The Mann-Whitney U test was used in case the data was not normally distributed. Univariate receiver operating characteristic (ROC) curve analysis was performed for analytes with the most significant differences (according to the *p*-value) and the area under the curve (AUC) was assessed. It enabled the comparison of the predictive value and the determination of sensitivity and specificity for selected amino acids. Sensitivity (true positive rate) corresponds to the percentage of positives correctly identified as positive, as opposed to specificity (true negative rate) which means negatives were properly classified as negative outcomes. The AUC was used to assess the efficiency of potential biomarkers. The higher the AUC value, the greater the probability of correct prediction of the outcome by the proposed model, where 1.00 means 100% of sensitivity and 100% of specificity. In our analysis we recognized the AUC ≥ 0.75 as significant. To analyze the differences between the three groups of patients the one-way ANOVA with Fisher’s LSD post-hoc analysis was performed.

Multivariate analysis: PLS-DA and DFA were preceded by normalization by sum, logarithm transformation, and auto scaling, to deal with non-normal data distribution and minimize the chance of skewed results by differences in concentration ranges between the variables (amino acid concentrations). Then the VIP score was assessed. This enabled the determination of the most important variables from PLS-DA. Forward stepwise DFA with post-hoc classification matrices was performed to evaluate the sensitivity and specificity of the discriminant models. In all tests a *p*-value less than 0.05 was regarded as significant.

To perform pathway analysis several open-source databases were used: Reactome (http://www.reactome.org/, date of visit: 3 October 2017), KEGG PATHWAY Database (http://www.genome.jp/kegg/pathway.html/, date of visit: 3 October 2017) and The Small Molecule Pathway Database (http://smpdb.ca/, date of visit: 3 October 2017) [32,33]. For data visualization draw.io—free online diagram software and MetaboAnalyst web serwer [31] (www.metaboanalyst.ca) were used.

Two types of comparisons were made to assess the diagnostic utility of amino acids in OC screening: (1) OC vs. HC; and (2) OC vs. BOT combined with HC. In the first analysis we identified amino acids that were altered in OC patients and that could be possibly considered OC biomarkers. The second analysis aimed at excluding the amino acids that could be altered by presence of BOT. It was proposed to reflect the clinical reality of the test used in screening assuming that in the screened population there are also women with benign ovarian pathologies and that a good test should be able to detect OC patients among all others.

## 5. Conclusions

The findings of our study provided new evidence on amino acid profile alterations in OC, broadening the knowledge about metabolic dysregulation in cancerogenesis and suggesting that serum-free amino acid profiling could be useful in the diagnosis and screening of OC. The performed statistical analyses identified histidine and citrulline as the most significantly altered amino acids in OC compared to BOT and HC. These two amino acids were indicated as promising targets for further evaluation as potential OC biomarkers.

## Figures and Tables

**Table 1:** Discriminant function analysis parameters for ovarian cancer (OC) versus healthy control (HC) group and OC versus combined benign ovarian tumor (BOT) and HC group.

Type of Comparison	Model 1	Model 2
	OC vs. HC	OC vs. (BOT + HC)
Compounds in the model	histidine, ornithine	histidine, citrulline, alanine, asparagine, ornithine
Wilks’ Lambda	0.63367	0.57495
*p*-value	*p* < 0.0001	*p* < 0.0001
Sensitivity (%)	76.32	60.53
Specificity (%)	80.00	94.64
Total Group Membership Classification (%)	78.41	86.00

**Table 2:** Review of recent metabolomic studies on OC biomarkers.

Authors	Biological Matrix	Groups	Technique	Metabolites/Groups of Metabolites Identified as Potential Biomarkers
Zhou, M., 2010 [14]	Human serum	44 serous papillary ovarian cancers, 50 controls	DART/TOF-MS	Metabolites involved in: amines and amino acids metabolism, eicosanoids
Zhang, T., 2012 [7]	Human plasma	80 epithelial ovarian cancers, 90 benign ovarian tumors	UPLC-QTOF-MS	Tryptophan, lysoPC(18:3), lysoPC(14:0), 2-piperidinone
Zhang, T., 2013 [19]	Human urine	40 preoperative epithelial ovarian cancers, 62 benign ovarian tumors, 54 healthy controls	UPLC-QTOF-MS	22 metabolites involved in: nucleotide metabolism, histidine metabolism, tryptophan metabolism, mucin metabolism
Ke, C., 2014 [15]	Human plasma	40 epithelial ovarian cancers, 158 benign ovarian tumors, 150 uterine fibroids	UPLC-QTOF-MS	53 metabolites involved in: phospholipid metabolism, tryptophan catabolism, fatty acid b-oxidation, metabolism of piperidine derivatives
Gaul, D.A., 2015 [20]	Human serum	46 early-stage (I/II) ovarian cancers, 49 healthy controls	UPLC-MS/MS	16 metabolites involved in lipids and fatty acids metabolism (phospholipids, lysophospholipids, sphingolipids)
Buas, F., 2016 [8]	Human plasma	50 serous ovarian cancers, 50 controls	LC-QTOF-MS; LC-MS/MS	Global lipidomics: 34 metabolites (glycerophospholipids, glycerolipids, sphingolipid, sterol lipid) Targeted profiling: alanine
Bachmayr-Heyda, A., 2016 [13]	Preoperative and follow-up sera, ascites, and tumor tissues	65 high-grade serous ovarian cancers, 62 healthy controls	LC-MS/MS	43 glycerophospholipids, 5 amino acids
Fan, L., 2016 [5]	Human plasma	21 early stage epithelial ovarian cancers, 31 healthy controls	UPLC-QTOF-MS	18 metabolites including lysophospholipids, 2-piperidone, monoacylglycerol (18:2)
Hilvo, M., 2016 [16]	Human serum and tumor tissue	158 high-grade serous ovarian cancers, 100 controls with benign or non-neoplastic lesions	GCxGC-TOF-MS	Tryptophan, 3-hydroxybutyric acid, 3,4-dihydroxybutyric acid
Li, J., 2017 [6]	Human plasma	39 epithelial ovarian cancer recurrent patients, 31 non-recurrent patients	UPLC-QTOF-MS	31 lipid metabolites including phosphatidylcholines, lysophosphatidylcholines, phosphatidylinositols

## Supplementary Materials

Supplementary materials can be found at: www.mdpi.com/1422-0067/18/12/2727/s1.

## Abbreviations

The following abbreviations are used in this manuscript:

OC	Ovarian cancer
FIGO	International Federation of Gynecology and Obstetrics
CA125	Cancer antigen 125
HE4	Human epididymis protein 4
BOT	Benign ovarian tumor(s)
HC	Healthy control(s)
LC-MS/MS	Liquid chromatography/tandem mass spectrometry
MRM	Multiple reaction monitoring
LOQ	Limit of quantitation
ROC	Receiver operating characteristic
AUC	Area under the curve
CI	Confidence interval
PLS-DA	Partial least squares-discriminant analysis
VIP	Variable importance in projection
DFA	Discriminant function analysis
HPLC-MS/MS	High-performance liquid chromatography/tandem mass spectrometry
DART/TOF-MS	Direct analysis in real time mass spectrometry
UPLC-QTOF-MS	Ultra-performance liquid chromatography-quadrupole time-of-flight mass spectrometry
UPLC-MS/MS	Ultra-performance liquid chromatography-tandem mass spectrometry
LC-QTOF-MS	Liquid chromatography-quadrupole time-of-flight mass spectrometry
GCxGC-TOF-MS	Two-dimensional gas chromatography with time-of-flight mass spectrometer
IDO	Indoleamine-(2,3)-dioxygenase
PRPP	5-Phosphoribosyl-1-pyrophosphate
HFBA	Heptafluorobutyric acid
