# Supplementary material for: Usefulness of Amino Acid Profiling in Ovarian Cancer Screening with Special Emphasis on Their Role in Cancerogenesis
Source: Int J Mol Sci. 2017 Dec 16;18(12):2727. doi: 10.3390/ijms18122727 (PMC5751328; doi:10.3390/ijms18122727)
Supplement: Supplementary file 1 [file ijms-18-02727-s001.docx]

**Supplementary Materials**

**Table S1.** The results of univariate statistical analyses based on determined amino acid concentrations for ovarian cancer (OC), benign ovarian tumor (BOT), and healthy control (HC) patients.

| **Amino acids** | **Concentration in serum [µM]** | | | | | | | | | **Type of comparison** | | | |
| --- | --- | --- | --- | --- | --- | --- | --- | --- | --- | --- | --- | --- | --- |
|  | **OC**  **(n = 38)** | | | **HC**  **(n = 50)** | | | **Combined BOT and HC**  **(n=112)** | | | **OC vs HC** | | **OC vs (BOT+HC)** | |
|  | **Mean** | **Median** | **SD** | **Mean** | **Median** | **SD** | **Mean** | **Median** | **SD** | **fold change** | ***p*-value^1^** | **fold change** | ***p*-value^1^** |
| 1-Methyl-L-histidine | 6.70 | 4.20 | 6.13 | 11.60 | 7.35 | 12.42 | 8.29 | 4.90 | 9.63 | **0.58** | **0.0345** | 0.81 | 0.7771 |
| 3-Methyl-L-histidine | 4.29 | 3.95 | 1.87 | 5.19 | 4.40 | 3.25 | 4.54 | 4.00 | 2.39 | 0.83 | 0.0776 | 0.95 | 0.4716 |
| α- Aminoadipic acid | 1.00 | 0.90 | 0.45 | 0.87 | 0.80 | 0.33 | 0.81 | 0.80 | 0.29 | 1.15 | 0.2735 | **1.24** | **0.0253** |
| α-Amino-n-butyric acid | 30.53 | 28.65 | 10.73 | 28.96 | 26.30 | 10.12 | 28.59 | 27.00 | 9.85 | 1.05 | 0.3563 | 1.07 | 0.2947 |
| Alanine | 396.79 | 375.75 | 122.32 | 444.69 | 413.10 | 106.46 | 429.04 | 410.95 | 96.27 | **0.89** | **0.0379** | 0.93 | 0.0625 |
| Arginine | 106.30 | 101.85 | 32.80 | 104.16 | 95.15 | 43.83 | 104.05 | 98.80 | 36.04 | 1.02 | 0.3810 | 1.02 | 0.5626 |
| Asparagine | 56.64 | 55.30 | 14.10 | 66.83 | 62.10 | 14.26 | 64.81 | 61.65 | 13.55 | **0.85** | **0.0026** | **0.87** | **0.0030** |
| Aspartic acid | 31.59 | 29.85 | 18.64 | 38.68 | 37.55 | 19.71 | 33.21 | 28.45 | 17.70 | 0.82 | 0.0741 | 0.95 | 0.6299 |
| β-Aminoisobutyric acid | 3.19 | 3.00 | 1.35 | 3.52 | 2.75 | 3.38 | 3.11 | 2.75 | 2.40 | 0.91 | 0.4688 | 1.03 | 0.2107 |
| β-Alanine | 38.33 | 32.00 | 54.76 | 27.80 | 27.80 | 11.76 | 28.20 | 28.55 | 10.61 | 1.38 | 0.3101 | 1.36 | 0.2530 |
| Citrulline | 22.22 | 21.25 | 7.71 | 29.88 | 29.10 | 7.11 | 29.40 | 28.25 | 6.78 | **0.74** | **<0.0001** | **0.76** | **<0.0001** |
| Cystine | 38.96 | 43.55 | 18.31 | 31.59 | 28.25 | 26.27 | 32.02 | 31.65 | 21.18 | **1.23** | **0.0454** | **1.22** | **0.0219** |
| Ethanolamine | 11.51 | 9.95 | 4.73 | 13.80 | 12.65 | 5.53 | 13.04 | 11.70 | 5.90 | **0.83** | **0.0473** | 0.88 | 0.2078 |
| Glutamine | 607.65 | 594.80 | 107.89 | 635.72 | 621.00 | 82.30 | 657.25 | 653.50 | 84.72 | 0.96 | 0.1696 | **0.92** | **0.0128** |
| Glutamic acid | 93.61 | 86.20 | 53.95 | 120.14 | 101.10 | 67.16 | 99.47 | 79.05 | 64.15 | 0.78 | 0.0632 | 0.94 | 0.9294 |
| Glycine | 305.11 | 283.05 | 91.72 | 326.16 | 294.50 | 102.14 | 321.01 | 296.45 | 95.45 | 0.94 | 0.3833 | 0.95 | 0.3827 |
| Histidine | 69.15 | 69.90 | 14.26 | 89.77 | 89.65 | 17.15 | 87.16 | 87.20 | 14.39 | **0.77** | **<0.0001** | **0.79** | **<0.0001** |
| Hydroxyproline | 11.41 | 9.60 | 7.85 | 11.47 | 9.95 | 5.68 | 11.16 | 9.90 | 5.11 | 0.99 | 0.6860 | 1.02 | 0.6207 |
| Isoleucine | 79.74 | 77.90 | 20.80 | 73.88 | 69.35 | 20.05 | 71.13 | 70.25 | 17.91 | 1.08 | 0.1152 | **1.12** | **0.0271** |
| Leucine | 133.33 | 131.30 | 37.62 | 133.89 | 126.70 | 33.08 | 125.53 | 123.45 | 30.80 | 1.00 | 0.9731 | 1.06 | 0.2888 |
| Lysine | 226.01 | 231.65 | 52.97 | 265.10 | 245.60 | 71.41 | 252.90 | 245.90 | 60.04 | **0.85** | **0.0158** | **0.89** | **0.0271** |
| Methionine | 23.02 | 21.65 | 7.42 | 25.60 | 24.50 | 7.04 | 24.89 | 24.30 | 6.07 | **0.90** | **0.0419** | **0.93** | **0.0412** |
| Ornithine | 109.36 | 101.45 | 45.18 | 139.44 | 137.90 | 46.69 | 127.02 | 122.80 | 48.64 | **0.78** | **0.0018** | **0.86** | **0.0393** |
| O-Phosphoethanolamine | 2.13 | 1.50 | 2.24 | 2.23 | 1.60 | 1.87 | 2.11 | 1.60 | 1.77 | 0.96 | 0.6074 | 1.01 | 0.5933 |
| Phenylanalanine | 76.23 | 75.65 | 17.54 | 79.26 | 76.05 | 19.39 | 73.97 | 72.00 | 17.32 | 0.96 | 0.4504 | 1.03 | 0.3676 |
| Proline | 184.31 | 187.50 | 63.79 | 173.76 | 157.50 | 54.65 | 179.72 | 171.45 | 56.07 | 1.06 | 0.2383 | 1.03 | 0.4613 |
| Sarcosine | 1.15 | 0.70 | 2.36 | 0.77 | 0.70 | 0.45 | 0.92 | 0.70 | 0.88 | 1.49 | 0.8431 | 1.25 | 0.6071 |
| Serine | 161.86 | 151.85 | 46.85 | 173.32 | 169.70 | 40.36 | 170.24 | 170.40 | 38.81 | 0.93 | 0.1022 | 0.95 | 0.1173 |
| Taurine | 190.60 | 181.35 | 85.86 | 195.97 | 208.70 | 62.38 | 183.43 | 184.65 | 60.03 | 0.97 | 0.7347 | 1.04 | 0.5719 |
| Threonine | 114.28 | 108.20 | 38.81 | 131.06 | 125.45 | 33.13 | 131.98 | 125.75 | 33.98 | **0.87** | **0.0186** | **0.87** | **0.0058** |
| Tryptophan | 41.31 | 42.90 | 11.03 | 48.68 | 47.50 | 10.56 | 48.97 | 49.45 | 9.37 | **0.85** | **0.0020** | **0.84** | **0.0001** |
| Tyrosine | 60.01 | 59.10 | 21.70 | 58.11 | 55.60 | 14.59 | 56.98 | 55.60 | 13.47 | 1.03 | 0.7015 | 1.05 | 0.5253 |
| Valine | 238.34 | 238.25 | 49.05 | 239.14 | 234.40 | 55.58 | 231.38 | 231.25 | 50.28 | 1.00 | 0.6462 | 1.03 | 0.4129 |

^1.^Bold type for *p*-values indicates statistical significance

**Table S2.** Demographic and clinical characteristics of the patients.

| **Characteristics** | **OC** | **BOT** | **HC** |
| --- | --- | --- | --- |
| No. of subjects | 38 | 62 | 50 |
| Age [years] |  |  |  |
| mean | 59.1 | 42.3 | 51.9 |
| median | 60 | 40.5 | 56 |
| range | 32-78 | 18-72 | 19-73 |
| Weight (mean) [kg] | 67.6 | 68.9 | 69.6 |
| BMI (mean) [kg/m^2^] | 26.5 | 25.6 | 26.8 |
| Menopause |  |  |  |
| pre- | 8 | 46 | 20 |
| post- | 30 | 16 | 30 |
| FIGO |  |  |  |
| I | 10 | - | - |
| II | 2 | - | - |
| III | 25 | - | - |
| IV | 1 | - | - |
| Histological type |  |  |  |
| Serous | 16 | - | - |
| Endometrioid | 4 | - | - |
| Mucinous | 1 | - | - |
| Clear cell | 3 | - | - |
| Undifferentiated | 10 | - | - |
| Other/unclassified | 4 | - | - |

**
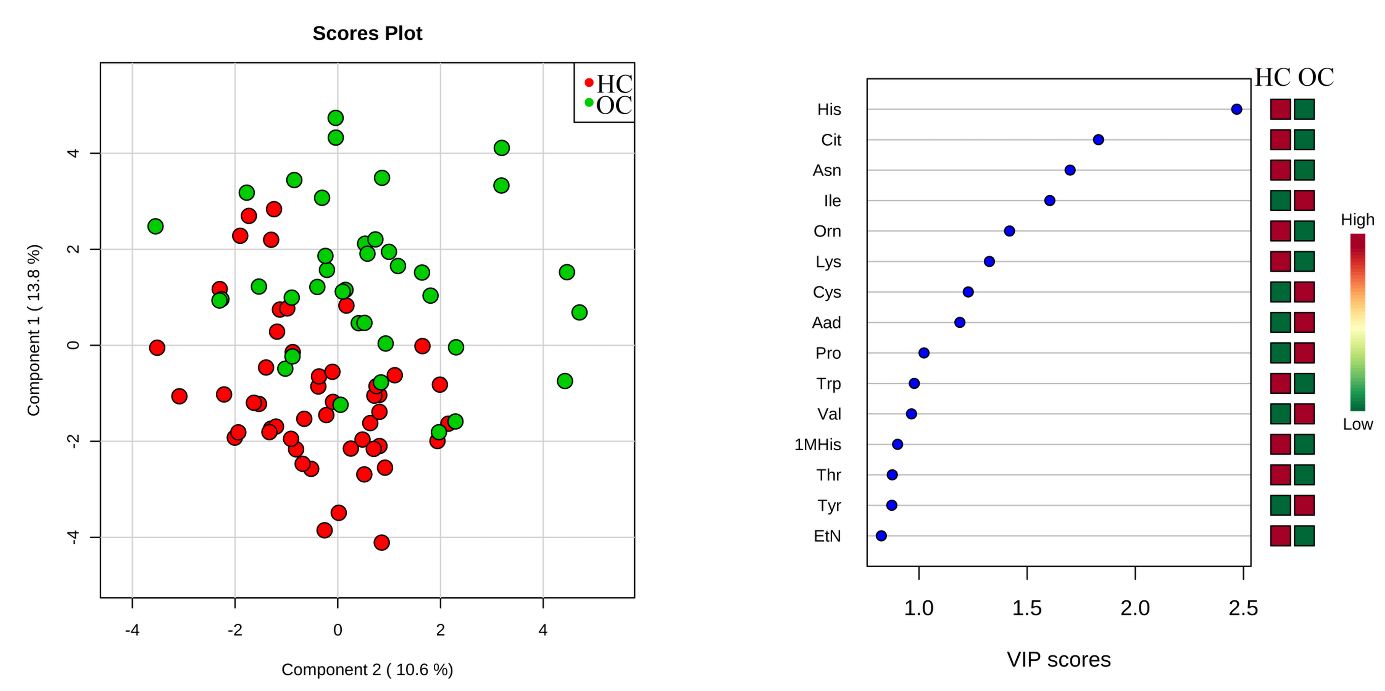
Figure S1.** PLS-DA score plot distinguishing OC from HC and the 15 amino acids with the highest VIP score values.

**
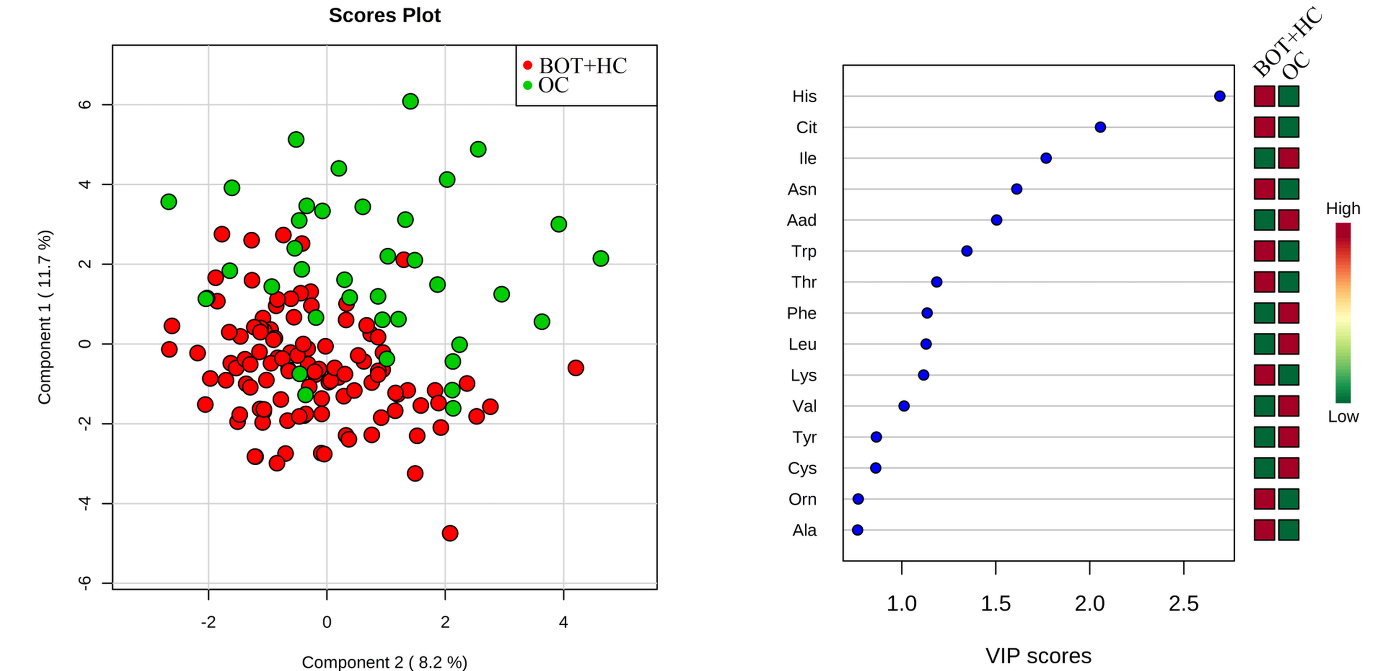
Figure S2.** PLS-DA score plot distinguishing OC from combined BOT and HC group and the 15 amino acids with the highest VIP score values.
